# Robustness to noise of arterial blood flow estimation methods in CT perfusion

**DOI:** 10.1186/1756-0500-7-540

**Published:** 2014-08-18

**Authors:** Maria Romano, Michela D’Antò, Paolo Bifulco, Francesco Fiore, Mario Cesarelli

**Affiliations:** DIETI, University of Naples, “Federico II”, Naples, Italy; Interuniversity Centre of Bioengineering of the Human Neuromusculoskeletal System, Rome, Italy; National Cancer Institute “Pascale Foundation”, Naples, Italy

**Keywords:** Computed tomography (CT), Liver perfusion, Maximum slope method, Dual-input one-compartment model, Noise robustness

## Abstract

**Background:**

Perfusion CT is a technology which allows functional evaluation of tissue vascularity. Due to this potential, it is finding increasing utility in oncology. Although since its introduction continuous advances have interested CT technique, some issues have to be still defined, concerning both clinical and technical aspects. In this study, we dealt with the comparison of two widely employed mathematical models (dual input one compartment model – DOCM - and maximum slope – SM -) analyzing their robustness to the noise.

**Methods:**

We carried out a computer simulation process to quantify effect of noise on the evaluation of an important perfusion parameter (Arterial Blood Flow – BFa) in liver tumours. A total of 4500 liver TAC, corresponding to 3 fixed BFa values, were simulated using different arterial and portal TAC (computed from 5 real CT images) at 10 values of signal to noise ratio (SNR). BFa values were calculated by applying four different algorithms, specifically developed, to these noisy simulated curves. Three algorithms were developed to implement SM (one semiautomatic, one automatic and one automatic with filtering) and the last for the DOCM method.

**Results:**

In all the simulations, DOCM provided the best results, i.e., those with the lowest percentage error compared to the reference value of BFa. Concerning SM, the results are variable. Results obtained with the automatic algorithm with filtering are close to the reference value, but only if SNR is higher than 50. Vice versa, results obtained by means of the semiautomatic algorithm gave, in all simulations, the lowest results with the lowest standard deviation of the percentage error.

**Conclusions:**

Since the use of DOCM is limited by the necessity that portal vein is visible in CT scans, significant restriction for patients’ follow-up, we concluded that SM can be reliably employed. However, a proper software has to be used and an estimation of SNR would be carried out.

## Background

Quantitative measurements of hepatic perfusion can give important information in detection, assessment and management of various liver diseases. Particular important is the measurement of blood flow within the liver, since changes in tumour vascularisation are significant indicators of treatment response of hepatic cancers [1]. Different methods of quantification have been proposed but generally are either invasive or remain controversial [2]. In the last decade, this awareness, the introduction of multidetector CT systems (MDCT) and the availability of perfusion commercial software have stimulated the clinical interest in perfusion CT technique [3]. This technique consists of sequential acquisitions of images during the intravenous injection of a contrast agent bolus. It provides parameters correlated to tumour vasculature and represents an in vivo marker of tumour angiogenesis [4, 5].

Hepatocellular Carcinoma (HCC), the most common malignant liver tumour, is characterized by an increased arterial vascularisation. An accurate assessment of arterial perfusion is then crucial to evaluate HCC response to treatments. Besides, primitive liver tumour diagnosis, assessment and staging are critical because PET (Positron emission tomography), that represent the gold standard functional technique, is not a useful tool in the diagnosis and follow up of HCC, because metabolism of glucose in primitive liver tumour is not different from the surrounding liver parenchima. So, liver perfusion CT studies are increasingly advocated as a means to assess the grade of vascularisation in HCC patients and to evaluate variations in perfusion parameters following locoregional treatments or antiangiogenic drugs. However, although since its introduction continuous advances have interested CT technique, in its use some problems remain still open. They concern both clinical and technical aspects, related, for example, to radiation dose, optimum volume and speed of bolus of contrast material injected, characteristics of the employed CT system and image processing [6–9].

About models to be adopted in assessment of perfusion parameters, currently three models are the most used, the maximum slope (SM) method, the dual-input one-compartment model (DOCM) method and the deconvolution method (DC) [8]. The SM method was introduced by Miles et al. [10] and, because its underlying principle is relatively simple, it has come to dominate the field of hepatic perfusion measurement. In contrast, this method can underestimate hepatic perfusion, especially portal perfusion, when the “no venous outflow” assumption is violated [11]. This assumption states that washout of contrast medium should not occur prior to the peak time of the initial slope of the tissue time attenuation curve (TAC). Thus, a high injection rate of contrast medium is a prerequisite for accurate perfusion measurements. To overcome this drawback, a new method of perfusion analysis, the DOCM method, was proposed by Materne et al. [2]. In theory, hepatic perfusion can be estimated correctly with this method regardless of the injection rate. Nevertheless, its use is time-consuming and limited by the necessity to include in the images also the portal vein. Cuenod et al. [12] used a deconvolution technique to evaluate hepatic perfusion. This method provides more robust analysis without a high injection rate, and the estimated perfusion values are theoretically independent from cardiac output, from possible delay of bolus or from other extrahepatic factors such as age or sex [8]. However, the calculation is complex and, mainly, the results are affected by the hemodynamic model used, which makes it unsuitable for the liver [8].

Despite to some encouraging results obtained with these models, there is currently no agreement regarding the optimal analytic method in hepatic CT perfusion and standardization in the use of model is still an open issue.

Recently, different authors [8, 13] have dealt with this topic. These studies have shown that no consensus has been reached about the choice of the best model; besides, they do not consider important aspects such as image noise. Noise in perfusion images is related to different aspects. A greater number of images results in more data points on the time attenuation curve (TAC), and therefore higher reliability of perfusion measurements. Similarly, a larger tube current results in less photon noise within each image. Image noise can also be reduced by using thicker image slices and lower resolution reconstruction filters but at the expense of spatial resolution [3]. Anyway, once the protocol is defined, there is always an unavoidable amount of noise which could heavily affect perfusion parameters estimation.

In this work, we focused on the comparison of the two most employed mathematical models (DOCM and SM) analyzing their robustness to the noise by means of computer simulations; in particular we quantified the effect of noise on evaluation of an important perfusion parameter (Arterial Blood Flow – BFa) in HCC lesions.

## Methods

### Patients

Five subjects (4 women and 1 man; age range, 70 – 77 years; mean, 74.2 years) with multiple or single hypervascular HCC lesions and without cardiac complications were enrolled for this study, by choosing in our database the images in which portal vein was visible. The diagnosis of HCC tumour was achieved on the basis of AASLD (American Association for the Study of Liver Disease) criteria using established techniques (RM, MDCT and CEUS) or by means of liver biopsy. Other relevant clinical information and weights were collected for all patients. A target untreated lesion was selected on the basal CTscan (without contrast). Then, a perfusion CT study was performed for each patient.

The project was approved by the scientific technical committee of the hospital (National Cancer Institute “Pascale Foundation”, Naples, Italy) as part of an internal research project with note DSC/1957 of 2009 and all patients gave informed consent to undergo investigation.

### CT perfusion imaging

Perfusion CT was performed by means of a commercially available scanner (Philips Brilliance 16 slices). The perfusion protocol comprised 30 scans (90 kVp, 250 mAs, 4 × 6 mm slice thickness, 1 second gantry rotation time, 3 s acquisition time), which were obtained in correspondence of tumour lesion. A 70 ml bolus of contrast agent (Iomeron 400 mg/ml) was injected (injection rate 4 ml/s) into an antecubital vein at the beginning of the CT data acquisition. The participants were advised to breathe slowly during the examination to reduce motion artifacts.

Images were exported for successive analysis by means of DICOM protocol and then were processed off-line using Matlab version 7.12.0.

### Mathematical methods

#### Maximum slope (SM)

The principle of the SM is quite simple, which made it a very attractive method. It is a derivation of Fick principle allowing the separate evaluation of dual liver blood supply component, i.e. arterial blood flow (BFa) and portal blood flow (BFp). BFa is calculated like the maximum slope of the liver TAC in its early phase divided by peak aortic attenuation (Eq.1). The time of peak splenic enhancement is used as cut-off point to separate arterial and portal components. BFp is calculated by dividing the maximum slope of the liver TAC in its late phase (after peak splenic) by peak aortic attenuation [5, 11]. Because advanced HCC is practically not nourished by portal blood flow, blood flow in the tumour consists almost exclusively of arterial flow alone [14, 15], therefore BFa is here chosen as perfusion parameter. In the following equation (1), C_l_(t) is TAC of tumour or liver parenchyma and C_a_(t) is aorta TAC.
1

Although its simplicity, the application of this method involves the choice of different processing techniques. In literature [16–18], different TAC processing have been analyzed and different algorithms have been developed for the application of this method but details are generally missing and no consensus has been reached about the most reliable algorithm to compute the maximum slope [16]. In particular, in literature it is not defined a method to identify starting and end points of the up-slope calculation [16].

### Dual-input one compartment model (DOCM)

With this method, hepatic perfusion parameters are calculated using all TAC points [19]. When this model is used, the differential equation describing the kinetic behaviour of the contrast agent is [1, 2, 13]:
2

where C_l_ (t), C_a_ (t) and C_p_ (t) are the concentrations of contrast agent at time *t* (TAC in the region of interest – ROI – ) in the liver, hepatic artery and portal vein, respectively. τ_a_ and τ_p_ represent the transit time of contrast agent respectively from the aorta and portal vein ROI, to the liver ROI. The minus signs before τ_a_ and τ_p_ occurs because the arrival times of the contrast agent to the liver ROI through the hepatic artery and portal vein are generally delayed compared with those to the aorta and portal vein ROI respectively. The parameters k_1a_, k_1p_ and k_2_ are the rate constants for the transfers of the contrast agent from the hepatic artery to the liver, from the portal vein to the liver, and from the liver to the blood.

### Curves simulation

In order to compare different BFa estimation methods and to test their robustness to noise, we carried out a computer simulation process.

We computed noise-free C_l_(t) starting from equation 2. To solve this equation, we used the linear least-squares method, according to Murase [1], with the assumption that the initial conditions are zero [1]. Besides, we fixed k_1a_, k_1p_ and k_2_ values and used a discrete trapezoids method for the integration.

Holding the hypothesis that in HCC only the arterial flow contribution is significantly changed, as introduced in the above section “Maximum slope”, values relative to healthy subjects were chosen for k_1p_ and k_2_ (respectively, 0.0133 and 0.0333 [1, 13]).

k_1a_ was fixed on the basis of a previous work about HCC [6] (please, see the next Section).

C_a_(t) and C_p_(t) were obtained from real perfusion CT, drawing circular ROI as large as possible on patients’ images.

Finally, τ_a_ and τ_p_ were assumed to be equal to zero for simplicity [13].

**Figure 1:**
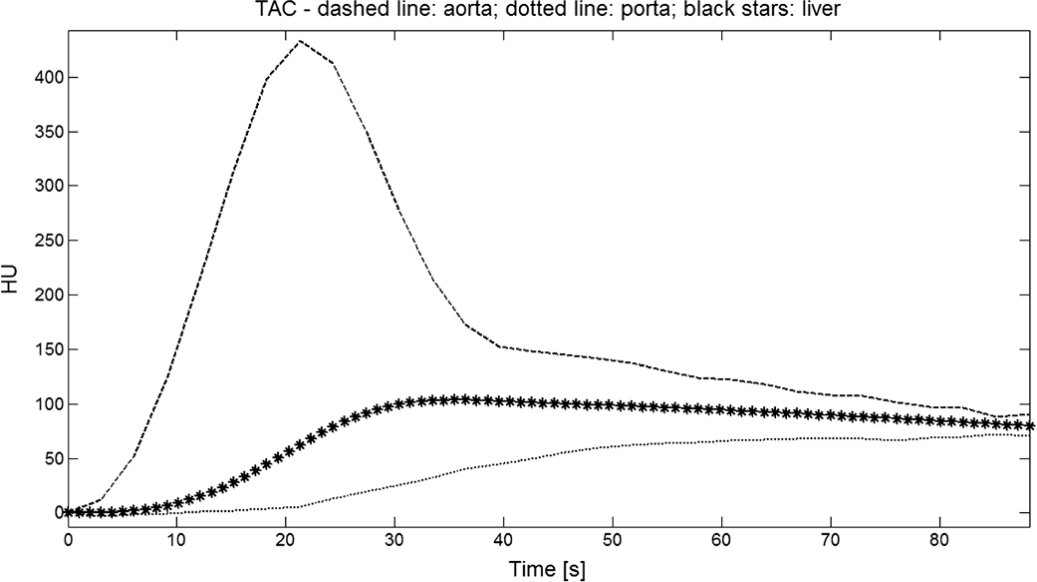
**Example of TAC used for the study.** An example of TAC used for this study (patient # 1, internal numbering). Dashed and dotted lines represent respectively aorta and portal TAC obtained from real perfusion images. Black stars mark a C_l_(t) (tumour TAC) estimated with the simulation study.

In Figure 1 an example of the curves used for the study is shown.

### Noise simulation

To investigate the effect of noise on BFa estimation, we added noise to the simulated C_l_(t). Specific models of noise should be adopted for the images here treated, but there is no literature available about this particular topic. Models employed in some research works about perfusion or described in other medical applications generally assume the noise to be additive, white and Gaussian [1, 13, 20]. So, we computed the noise by generating normally distributed random numbers with null mean and unit variance. Nevertheless, short sequences obtained by Matlab noise generator could not have unit variance. In order to ensure the unit variance of the noise, the generated random sequence (for simplicity called rum) was normalised with respect to its standard deviation. Then, in order to obtain set signal to noise ratio (SNR), we multiplied rum by the square root of the ratio between signal power and the set SNR, according to formula 3:
3

where P(signal) is the power of the simulated C_l_(t) curve.

**Figure 2:**
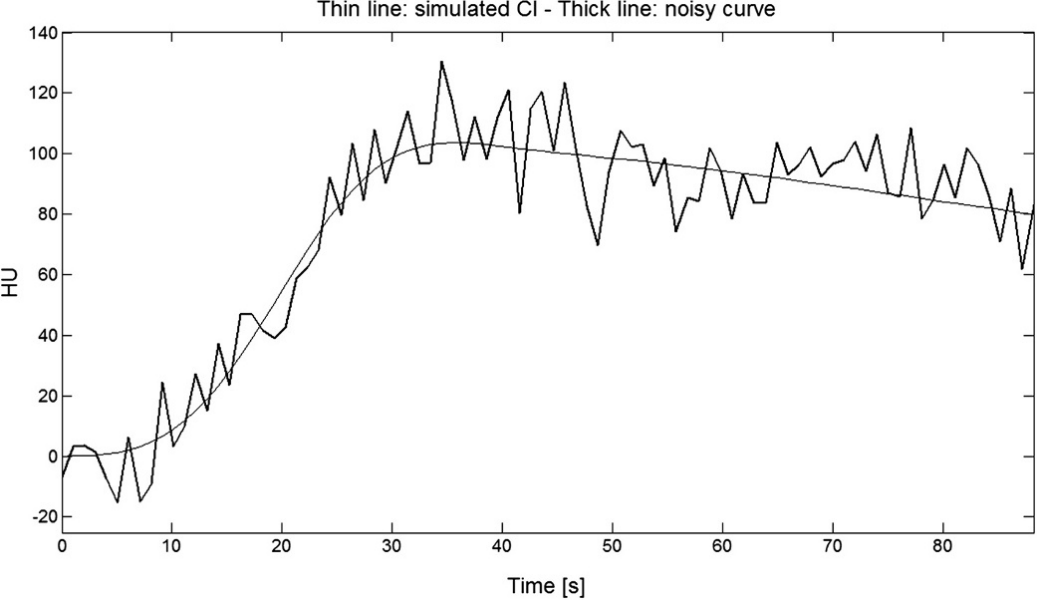
**Simulated Cl and noisy curves.** The thin line is the simulated C_l_ TAC shown in Figure 1 and the thick line is the same curve with added a noise signal such has to have SNR = 50*.*

In Figure 2 it is shown an example of a simulated C_l_ TAC and the corresponding noisy curve.

In order to obtain curves with different levels of noise, SNR ranged from 10 to 100 (step 10, absolute values). For each SNR value, the noise simulations were repeated thirty times; so that we obtained 300 C_l_ noisy curves for each C_l_(t).

### Simulation study

The procedure described in the previous Section (corresponding to 300 simulations with the same C_l_ curve and different signal noises, in the following also called patient study) was reproduced for each of the five patients (using the corresponding real C_a_ and C_p_ curves).

Then, the whole simulation study (involving all patients, i.e. 1500 simulations) was repeated three times, with different values of K_1a_ (for a total of 4500 simulations).

In particular, as values for k_1a_ were chosen 0.0146, 0.0096 and 0.0189. They were computed starting from mean, minimum and maximum values of BFa experimentally obtained in a previous work [6], making the appropriate change of measurement units and rounding to fourth decimal digit.

### BFa estimation

To use known perfusion units (ml × min^−1^× 100 ml^−1^), according to literature, all perfusion values were multiplied by 60 s/min and by 100 ml (of blood)/ml (of tissue), where we assumed a specific tissue gravity of 1.0 [11].

Experimental BFa values were estimated by applying four different algorithms (described below) to the noisy simulated C_l_ (t) curves. Three algorithms were developed to implement SM and the last for the DOCM method.

The algorithms performances were evaluated by comparing the obtained BFa with the set value.

The set BFa values, used as reference in the three simulation studies, were 87.6, 57.6 and 113.4 ml/min/100 ml (obtained multiplying by 6000 the k_1a_ values chosen for the simulations).

### SM semiautomatic algorithm

In this version, according to other algorithms proposed in literature [13], the algorithm is based on the manual selection of starting (S) and end (E) points of TAC range on which to compute the maximum slope [21, 22]. S and E points can be selected on C_l_ (t) by the operator through a simple interface, also developed with Matlab. The maximum slope was estimated as the slope of the straight line that fits the curve samples, between the two selected points, best in a least-squares sense.

### SM automatic algorithm

According to literature [16], the simulated C_l_ (t) curve was differentiated and an array (here named 1-D-diff), which represented the contrast time variations, was computed. At this point, the search of the maximum element was limited to the rise portion of the curve; in this case S was set equal to 10 s and E was fixed at one third of C_l_ length. Since, as mentioned, there are not references in literature, we based this choice on simulated curves computed by Bae [7] and on our experience. The largest element between S and E in the 1-D-diff array (which of course is positive) corresponds to the maximum contrast variation. Three consecutive data points of C_l_ curve, centred around the identified element, were then considered. The three consecutive points were fitted using a linear curve fitting model, the best fitting was again chosen minimizing the square mean error. The slope of the regression line was considered as the maximum slope of the TAC.

### SM automatic algorithm with smoothing

In this last version of the algorithm, as proposed in literature [17], we smoothed C_l_(t), before automatic computation of maximum slope, in order to reduce noise contribute. For smoothing, we applied an average filter (5th-order moving-average filter, cut-off frequency equal to 0.1 Hz).

### DOCM algorithm

In this case, to estimate the kinetic parameters and consequently BFa, we solved the eq. 2 using the same methodology described in the section “Curves simulation” but, of course, leaving the k values unknowns.

### Noise estimation

Assuming that the performances of the different signal processing techniques depend on SNR, we implemented a simple procedure for SNR estimation, to be applied on real curves in order to establish if the actual SNR permits the use of the proposed algorithms.

For assessing SNR, we computed the ratio between the estimates of signal power and noise power. In particular, the estimation of signal power was carried out calculating the power of the whole noisy C_l_(t) curve. Then, we estimated noise contribution as power of the curve in its final tract, where it is possible to assume that the contrast enhancement reached an almost steady-state plateau [7]; in this signal tract the computation of noise power is possible simply after removing any possible linear trend of the signal.

## Results

For simplicity, in the following, the acronyms listed in Table 1 will be used to indicate the results obtained.

**Table 1:** **Acronyms**

Acronym	Meaning
BFa_DOCM	BFa values computed with the DOC algorithm
BFa_SM_sa	BFa values obtained with the SM semiautomatic algorithm
BFa_SM_a	BFa values provided by the SM automatic algorithm
BFa_SM_af	Results of the SM automatic algorithm with filtering (i.e. after C_l_(t) smoothing)
Patient study	Simulations carried out for one patient and for all ten SNR values (for a total of 300 simulations)
SNR study	Simulations carried out for one SNR value and for all five patients (150 simulations)
Simulation study	1500 simulations (all patients and all SNR values)
BFa min	Simulation study (five patients, ten SNR values, thirty simulations, for a total of 1500 simulations) carried out with the reference BFa set at the min value (57.6)
BFa mean	Simulation study carried out with the reference BFa set at the mean value (87.6)
BFa max	Simulation study carried out with the reference BFa set at the max value (113.4)

Acronyms used to illustrate results.

**Figure 3:**
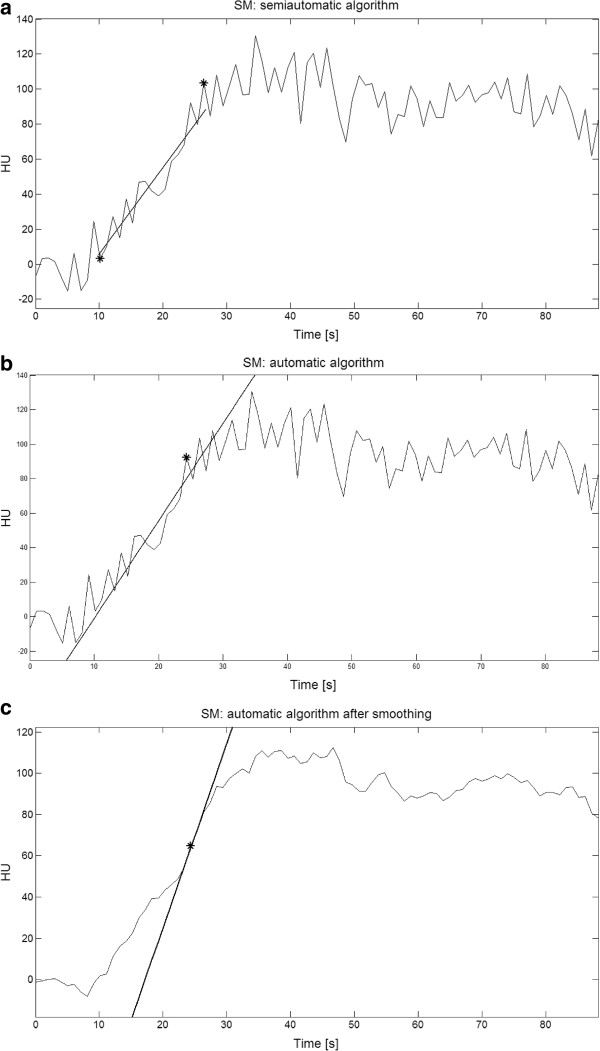
**Example of behaviour of the algorithms developed for implementation of slope method.** Behaviour of the algorithms developed for implementation of SM. Estimated regression lines are shown superimposed on a simulated noisy Cl curve (the same reported in Figure 2). From the top: **a)** semiautomatic algorithm, black stars represent start and end points selected from the operator; **b)** automatic algorithm; **c)** automatic algorithm after smoothing. In b and c the black stars represent the point automatically recognised from the algorithm as sample at maximum slope. In this case, computed BFa were respectively: 68.55, 78.30 and 123.81 ml/min/100 ml.

In Figure 3, to provide a clear, visual example of the software functioning, regression lines estimated from the three different algorithms which implement SM are shown superimposed on the simulated noisy C_l_ curve represented in Figure 2 (DOCM algorithm is not reported because of course it computes a value of BFa without the necessity of a regression line).

For the simulation shown in Figure 3 (one of the thirty simulations carried out for the patient #1 with SNR = 50 in the simulation study BFa max), BFa computed with DOCM algorithm was 116.90 (very close to the reference BFa) and the estimated SNR was 47.89.

The results obtained in the three simulation studies (different reference BFa values) are reported as mean ± standard deviation in Table 2. Each value of Table 2 was computed considering all five patients. The BFa values obtained in our simulation studies have shown a similar behaviour for all studied patients.

**Figure 4:**
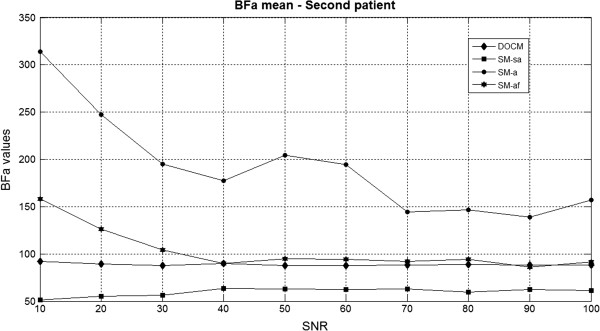
**BFa mean values (over 30 simulations) computed with BFa reference set to 87.6 (second patient).** BFa mean values (second patient) computed with the four algorithms for each set SNR (shown on the x-axis). Reference BFa was 87.6.

In Figure 4 are shown, as average values, the results of all thirty executions, at different SNR, corresponding to the second patient, obtained in the simulation study BFa mean.

**Table 2:** **BFa mean values obtained in the three simulation studies**

	DOCM	SM_sa	SM_a	SM_af
***BFa min (57.6)***	58.25 ± 6.71	39.63 ± 6.53	142.53 ± 109.90	78.01 ± 29.31
***BFa mean (87.6)***	88.69 ± 8.27	58.83 ± 10.53	192.67 ± 149.63	109.23 ± 41.24
***BFa max (113.4)***	113.66 ± 10.19	67.14 ± 16.70	208.54 ± 153.51	124.17 ± 39.63

Results obtained in the three simulation studies with the four algorithms (each pair of values corresponds to mean and standard deviation computed over 4500 simulations).

Observing Table 2 and Figure 4, it is possible to do some considerations (suitable for each condition – different patient and/or simulation study).

BFa values obtained with DOCM and automatic maximum slope algorithm with smoothing (columns named BFa_DOCM and BFa_SM_af) are the closest to the set value; though the automatic algorithm after filtering fails if SNR is at too low levels.

Manual selection of maximum slope (column BFa_SM_sa) leads always to underestimate BFa values, nonetheless results are little variable, as it results more clearly in Figure 4.

Finally, automatic algorithm (without any smoothing processing), because the great effect of the noise on C_l_(t), provided always the worst results (column BFa_SM_a), being the most variable and overestimating the set BFa in each simulation study and for each SNR value.

To better highlight differences between the estimated values of BFa and that set as reference (87.6 in this simulation study), we computed also the relative percentage errors (formula 4).
4

where BFa is the computed value and BFa_ref is the reference.

**Figure 5:**
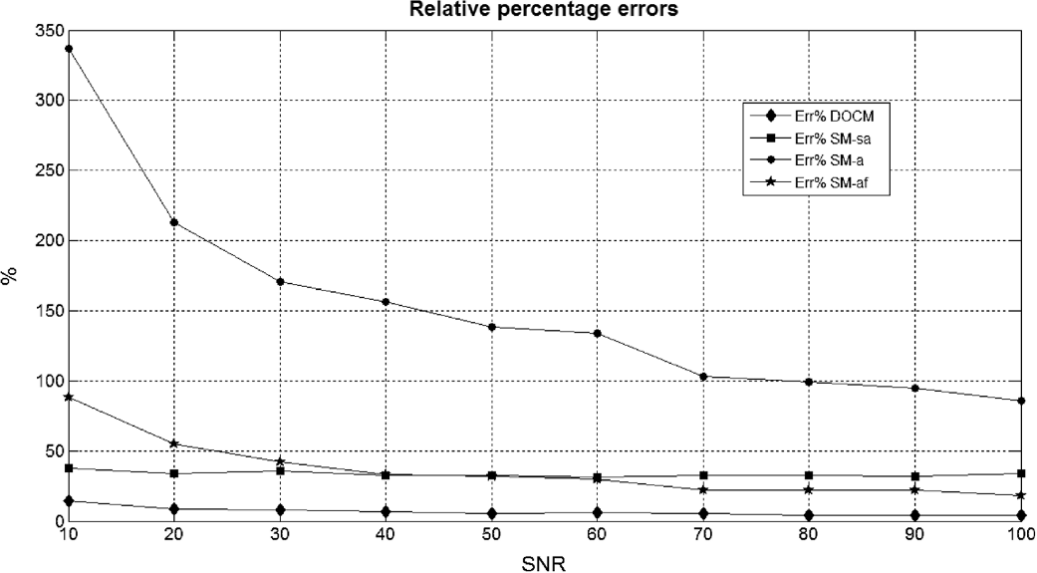
**Relative percentage errors relative to the different algorithms for each SNR study.** Relative percentage errors between estimated mean values of BFa and the reference one. Each point corresponds to one SNR study (SNR values are shown on the x-axis).

Values obtained for the simulation study with BFa equal to 87.6 are shown in Figure 5, grouped for each SNR study.

Mean and standard deviations of all these errors computed for the three simulation studies (BFa min, BFa mean, BFa max) are reported in Table 3, from which it is clear that the DOCM method on average makes the lowest error.

**Table 3:** **Relative percentage errors obtained on average for each simulation study**

	Err% DOCM	Err% SM_sa	Err% SM_a	Err% SM_af
***BFa min***	8.27 ± 8.28	31.39 ± 10.79	182.82 ± 157.19	44.28 ± 43.40
***BFa mean***	6.74 ± 6.73	33.26 ± 10.81	153.21 ± 141.72	36.51 ± 38.64
***BFa max***	6.48 ± 6.23	40.81 ± 14.67	114.14 ± 111.05	25.47 ± 25.74

For each simulation study (1500 simulations), mean ± standard deviation of relative percentage errors of BFa estimated with the four developed algorithms with respect to the set value.

In Table 4, instead, we reported the average value of the relative percentage errors for the automatic algorithm with filtering computed for each SNR study, since this parameter affects strongly the performance of this software.

**Table 4:** **Mean values (each by 150 simulations) of the Err% for the semiautomatic algorithm with filtering**

Err% SM_af			
SNR	***BFa min***	***BFa mean***	***BFa max***
**10**	98.95	88.45	61.77
**20**	65.05	55.16	34.80
**30**	53.22	42.27	29.29
**40**	42.88	33.22	23.98
**50**	39.06	31.82	20.35
**60**	32.14	29.56	18.64
**70**	31.88	21.94	17.92
**80**	29.24	22.28	18.97
**90**	23.45	22.38	15.67
**100**	26.90	17.99	13.32

Average value of the relative percentage errors computed for each SNR study, here indicated by bold numbers, and for the different set BFa (each value is the average of results obtained by 150 simulations).

Results reported in Table 4 indicate that the relative percentage error depends both on BFa value and SNR level.

Considering then the semiautomatic algorithm (SM_sa), it is possible to observe (Table 3 and Figure 5) that the standard deviation of % error ois quite low, regardless to SNR.

This result suggested us to compute a “modified” BFa value starting from that estimated with the semiautomatic algorithm and using a correcting factor (CF), computed as the overall mean percentage error, about equal to 35, as in formula 5.
5

In this way, starting from the estimations obtained for the different SNR studies (second, fourth and sixth column in Table 5), we obtained the BFa values reported in Table 6 (third, fifth and seventh column), which are much closer to the reference BFa value.

**Table 5:** **Mean BFa values obtained after correction (starting from results provided by the semiautomatic algorithm)**

SNR	***BFa min (57.6)***		***BFa mean (87.6)***		***BFa max (113.4)***	
	SM_sa	SM_sa_mod	SM_sa	SM_sa_mod	SM_sa	SM_sa_mod
**10**	37.97	58.41	57.82	88.96	64.99	99.98
**20**	38.96	59.93	58.68	90.27	68.08	104.75
**30**	38.90	59.84	56.26	86.55	65.56	100.87
**40**	40.14	61.75	59.30	91.23	67.71	104.17
**50**	39.11	60.17	59.29	91.21	64.46	99.17
**60**	39.58	60.90	60.70	93.39	66.25	101.92
**70**	40.63	62.50	59.18	91.05	68.27	105.03
**80**	40.45	62.22	59.15	91.01	67.00	103.07
**90**	40.70	62.62	59.68	91.81	69.09	106.30
**100**	39.91	61.40	58.23	89.58	70.04	10 7.75

BFa values obtained starting from estimations provided by the semiautomatic algorithm - SM_sa - and using the correcting formula - SM_sa_mod – (each value is the average of results obtained by 150 simulations), for each SNR study (bold numbers).

**Table 6:** **Mean estimation of SNR values**

SNR	***BFa min***	***BFa mean***	***BFa max***	Mean
**10**	11.18	11.43	11.66	**11.42**
**20**	21.62	21.80	21.61	**21.68**
**30**	30.97	30.63	31.84	**31.15**
**40**	40.51	42.33	40.89	**41.24**
**50**	48.48	49.69	50.63	**49.60**
**60**	58.29	60.64	63.84	**60.92**
**70**	67.98	69.11	71.13	**69.41**
**80**	76.10	77.83	82.48	**78.80**
**90**	85.95	86.08	94.22	**88.75**
**100**	91.80	96.11	101.91	**96.61**

Mean SNR values estimated with the proposed method. In bold set and overall mean SNR.

Finally, concerning SNR estimation, in Table 6 we reported the mean values obtained in the 150 simulations (30 for each patient) performed for each SNR study and for each simulation study.

It is possible to observe that set and estimated values, except in a few cases, are always very similar.

## Discussion

Diagnostic imaging techniques provide limited evaluations of tissue characteristics beyond morphology, whereas quantifying reliably angiogenesis is very important for evaluation of tumour progression and monitoring of the therapeutic response of HCC. CT perfusion has the potential to achieve this objective [23, 24]. This technique is quickly spreading in the field of hepatic oncology, since it is minimally invasive and can be quite simply incorporated into routinely CT protocols providing precious information about tumour grade and angiogenesis monitoring “in vivo” [5–7]. However, consistent, routine clinical application of perfusion CT requires a reliable employ of the technique. At the moment of research starting, there existed encouraging preliminary findings about reproducibility of the methodology and intra- and inter-observer variability but they did not regard the liver [25]. Besides, some aspects of crucial importance are still debated, for example effects of extra-hepatic factors [7] and standardization and validation of the analytic method to be employed [24], problem here faced. Moreover, although software packages involving perfusion parameters computation were addressed as advantageous in oncological applications [7], details of signal processing are lacking in literature so making difficult the procedures’ reproducibility.

Aim of this work was to compare two among the most diffused methods, SM and DOCM, testing three different algorithms for application of SM and one for DOCM and studying noise effect on the estimation of BFa, since, at the best of our knowledge, no study of this kind is available in literature. In fact, for example, Kanda et al. [8] have recently compared three analytic methods (SM, DOCM and DC, in their work named by different acronyms) but without considering noise contribution and Murase et al. [1] investigated effect of noise but only in the comparison of two computation methods for DOCM use.

Since there is no a practical method to evaluate in a reliable and accurate way perfusion parameters in vivo that can be considered the gold standard [11], we carried out a computer simulation process comparing estimated BFa with reference values.

In this study, according to literature, we considered a SNR range between 10 and 100. The lowest values characterise TAC computed using very small ROI or the pixel-by-pixel analysis typical in maps generation.

In all the 4500 simulations (30 for each of the 10 SNR values, 300 for each patient, 1500 for each simulation study), DOCM provided the best results, i.e., those with the lowest percentage error compared to the reference value of BFa (see Table 3).

About SM, its application by means of the semiautomatic algorithm provided always results lower than both the set BFa value and values estimated with the other methods, as shown in Figure 4. This is not so surprising, in fact other authors highlighted that perfusion parameters computed with SM are lower than those obtained with DOCM method, both in clinical studies and in simulation analysis [8, 11, 13], though, there, technical details about signal processing are not given. Furthermore, it is important to put in evidence that the semiautomatic algorithm computes the linear interpolation of the TAC between two points (start and end points) providing the estimation of its slope, and in turn an estimate of the average slope, while the automatic algorithm estimates the slope of the curve at its maximum slope point (overestimated in presence of noise). Of course, the mean slope value is surely lower than the maximum slope.

However, results got with the semiautomatic algorithm are quite stable, in the sense that they show a standard deviation of the percentage error (Table 3, column named Err% SM_sa) very low and not dependent on SNR. Hence, we propose to use a correcting factor (formula 4) to compute the BFa value starting from that in output of the software. Here, we imposed CF equal to 35 (mean percentage error), obtaining satisfactory results (please refer to Table 5); however, a deepened study, carried out on a more numerous set of images would be useful in order to set this parameter in a more general and reliable way.

Concerning the application of SM with automatic algorithm following a smoothing filtering, obtained results are not so far from the set value, confirming that it is useful to attenuate the irregularities of TAC before assessing perfusion parameters, as reported also in literature [17]. Nevertheless, this is valid only until SNR is over 50 (please refer to Figure 5 and Table 4), beyond this value the percentage error increases up to about 40%. Therefore, in order to apply this algorithm, is necessary to know the SNR of the image under analysis. Since, of course, in clinical practice this value is not known, we proposed a simple procedure for its estimation. As shown in Table 6, obtained results are satisfactory. So it could be possible to include this procedure in the image processing when an automatic algorithm is preferred.

Summarising, DOCM seems to be more reliable but it to be taken into account that for its use it is necessary that portal vein is visible in CT scans. This can be an important limitation, in fact portal vein is not always observable even using 64 slices CT. In our study, for example, only images recorded by five patients, in a database populated by 17 patients’ images, satisfy this requirement. The constraint is even more important for patients’ follow-up.

Reliability of patient follow-up evaluation is very important in medicine, and in particular in oncology, for assessment of therapy response of tumours [26]. Nevertheless, in the choice of the method, to be employed for estimation of perfusion parameters, no agreement has been reached and, according to Kanda et al. [8], it is not possible to interchange results obtained with different methods (SM, DOCM, DC). So that it can be crucial to employ always the same methodology for BFa assessment. In this case, our results suggest that SM can be reliably used but with attention to the particular processing employed.

## Conclusions

Perfusion CT, a technology which allows functional evaluation of tissue vascularity, is finding increasing utility in oncology and it is more and more often used as a means to assess the grade of vascularisation in HCC patients. Nevertheless, the best model to be adopted in assessment of perfusion parameters has not been yet established. On the basis of results shown in this work, and according with great part of literature, we found that DOCM provides the best assessment of perfusion, in particular of BFa, here estimated in a computer simulation process. However, the use of DOCM is limited by the necessity that portal vein is visible in CT scans, significant restriction mainly in patients’ follow-up, for which it is necessary to use always the same methodology. In these cases, we suggest that SM can be a useful and reliable alternative but a proper software for TAC processing has to be used and an estimation of SNR would be carried out before its use.
